# Repeated quantum error correction on a continuously encoded qubit by real-time feedback

**DOI:** 10.1038/ncomms11526

**Published:** 2016-05-05

**Authors:** J. Cramer, N. Kalb, M. A. Rol, B. Hensen, M. S. Blok, M. Markham, D. J. Twitchen, R. Hanson, T. H. Taminiau

**Affiliations:** 1 QuTech, Delft University of Technology, PO Box 5046, 2600 GA Delft, The Netherlands; 2 Kavli Institute of Nanoscience, Delft University of Technology, PO Box 5046, 2600 GA Delft, The Netherlands; 3 Element Six Innovation, Fermi Avenue, Harwell Oxford, Didcot, Oxfordshire OX11 0QR, UK

## Abstract

Reliable quantum information processing in the face of errors is a major fundamental and technological challenge. Quantum error correction protects quantum states by encoding a logical quantum bit (qubit) in multiple physical qubits. To be compatible with universal fault-tolerant computations, it is essential that states remain encoded at all times and that errors are actively corrected. Here we demonstrate such active error correction on a continuously protected logical qubit using a diamond quantum processor. We encode the logical qubit in three long-lived nuclear spins, repeatedly detect phase errors by non-destructive measurements, and apply corrections by real-time feedback. The actively error-corrected qubit is robust against errors and encoded quantum superposition states are preserved beyond the natural dephasing time of the best physical qubit in the encoding. These results establish a powerful platform to investigate error correction under different types of noise and mark an important step towards fault-tolerant quantum information processing.

## Abstract (web summary)

Large-scale quantum information processing requires the continuous protection of quantum states against errors. Here, the authors demonstrate active quantum error correction that improves the dephasing time of quantum states using a diamond quantum processor.

## Graphical abstract

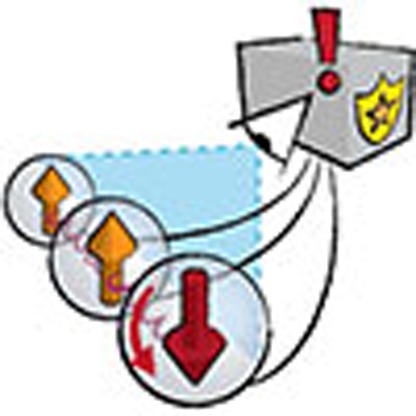

Large-scale quantum information processing requires the correction of errors during computations. In quantum error correction, a logical quantum bit (qubit) is encoded in a subspace of multiple physical qubits so that errors can be actively corrected without affecting the encoded information. A promising way to correct errors in encoded quantum states is to perform feedback based on multi-qubit measurements known as stabilizer measurements^1^,^2^,^3^ (see Fig. 1a for details). These measurements are performed non-destructively using extra qubits (ancillas) and are frequently repeated to detect errors before they accumulate. The measurement outcomes are then processed in classical logic that identifies the error syndrome, and, in order to enable universal computations^1^, active feedback is applied to the encoded system to correct errors where needed. The key experimental challenge is to perform such complete error-correction cycles including non-destructive stabilizer measurements and real-time feedback well within the coherence time.

Quantum-error-correction protocols have been explored across a range of platforms^4^,^5^,^6^,^7^,^8^,^9^,^10^,^11^,^12^,^13^,^14^. Pioneering experiments bypassed stabilizer measurements by reversing the encoding to correct errors, thus leaving the quantum state unprotected^5^,^6^,^7^,^8^,^9^,^10^,^11^. Recent breakthroughs have enabled the use of stabilizer measurements to passively track errors in quantum states and retrieve stored information afterwards through post processing^12^,^13^,^14^,^15^.

Here we realize complete rounds of active quantum error correction on a continuously encoded logical qubit by exploiting newly developed stabilizer measurements based on an electron spin ancilla with high-fidelity non-demolition readout, by encoding in long-lived nuclear spins, and by applying real-time correction of errors through fast classical logic. We show that the actively error-corrected logical qubit is robust against errors and that multiple rounds of error correction prevent errors from accumulating. Finally, by correcting time-correlated phase errors naturally induced by the environment, we demonstrate that encoded quantum superposition states are preserved beyond the dephasing time of the best physical qubit used in the encoding.

## Results

### Error correction code

The three-qubit code considered here corrects a single phase error on any one of the physical qubits. To protect against such errors, we encode the logical qubit in states for which all physical qubits have the same phase: 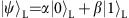 with 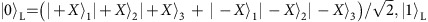

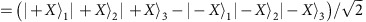
 and 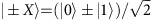. Errors (*Z* operations) are detected by measuring the two stabilizer generators *X*_1_*X*_2_*I*_3_ and *I*_1_*X*_2_*X*_3_ via an ancilla. These measurements, respectively, compare the phases of qubits 1 and 2 and qubits 2 and 3. For an uncorrupted state, both measurements yield outcome +1 (same phase, no error), but for a phase error on just one of the qubits, the two measurements give a unique syndrome of −1 outcomes that identifies the error. For example, an error on the first qubit results in outcome −1 for the first stabilizer measurement and outcome +1 for the second. The logical qubit operators are *X*_L_=*X*_1_*I*_2_*I*_3_, *Y*_L_=*Y*_1_*Z*_2_*Z*_3_ and *Z*_L_=*Z*_1_*Z*_2_*Z*_3_ (or their permutations).

### Stabilizer measurements and real-time feedback

**Figure 1:**
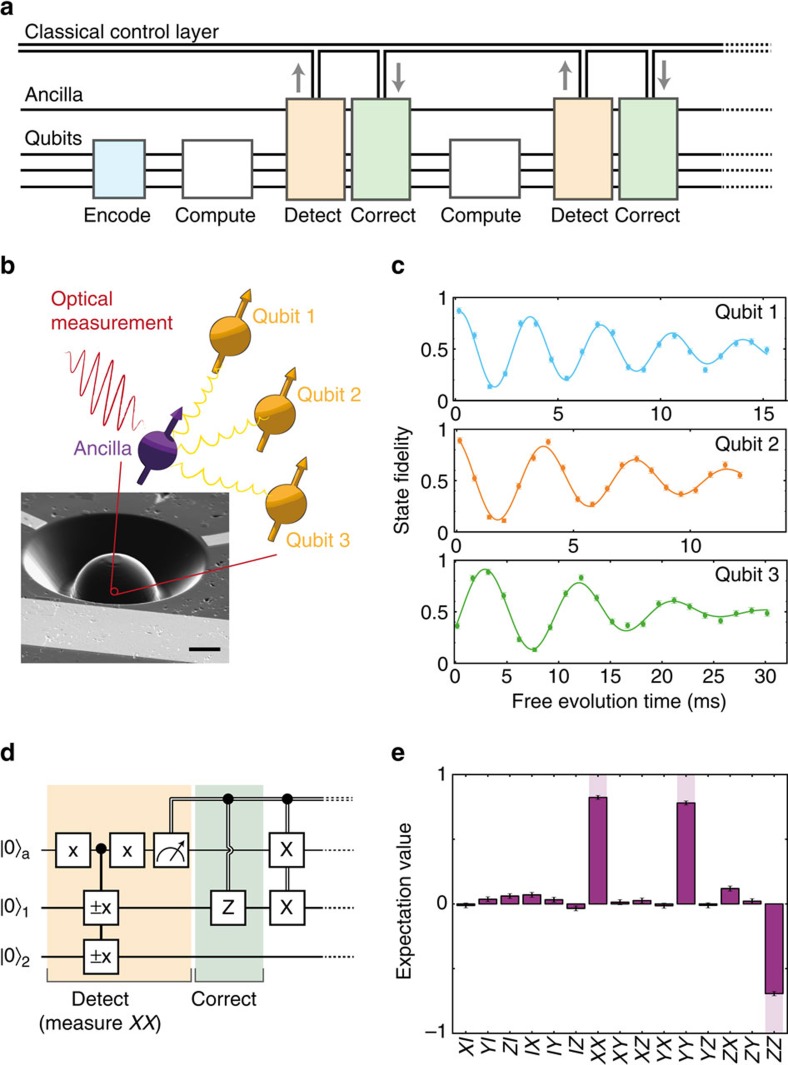
Quantum error correction and implementation of stabilizer measurements. (**a**) A quantum state is encoded in a logical qubit consisting of three physical qubits. Errors inevitably occur, for example, during computations. An ancilla is used to repeatedly perform measurements that detect errors. Errors are corrected through classical logic and feedback, while the quantum state remains coherent and encoded. (**b**) Device: chemical-vapour-deposition-grown single-crystal diamond with a solid-immersion lens^37^ and on-chip lines for microwave control. Scale bar, 5 μm. Ancilla: the optically addressable electronic spin of a nitrogen vacancy (NV) centre. Qubits: three ^13^C nuclear spins that are controlled and measured through the hyperfine coupling to the ancilla (Methods). (**c**) Free induction decay (Ramsey) experiments. Gaussian fits yield dephasing times 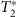
=12.0(9), 9.1(6) and 18.2(9) ms for qubits 1, 2 and 3, respectively. (**d**) Deterministic entanglement of two qubits by *XX* stabilizer measurement and feedback. The ±*x* gates are *π*/2 rotations around *x* with the sign controlled by the ancilla state. The final *X* operations reset the ancilla and account for an additional *X* flip for the +1 outcome (Methods). (**e**) State tomography of the generated entangled state for qubits 2 and 3. The fidelity with the ideal state is *F*=0.824(7) (see Supplementary Fig. 6 for other qubit combinations and post-selected results). All error bars are one statistical s.d.

Our qubits are three ^13^C nuclear spins (*I*=1/2, 1.1% abundance) surrounding a single nitrogen-vacancy (NV) centre in diamond, whose electronic spin we use as ancilla (*S*=1; 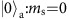 and 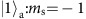; Fig. 1b). At 4 K, the ancilla combines fast control^16^, optical single-shot readout^17^ and long coherence times^18^ (>25 ms, Methods). We use relatively remote nuclear qubits (coupling to the ancilla 20–50 kHz) that are robust against optical excitation of the ancilla and design decoherence-protected gates to control them^9^,^19^ (Methods). All three qubits show long dephasing times 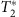 with the dominant natural errors being phase errors (Fig. 1c).

The key challenge for implementing stabilizer measurements in this system is that the ancilla–qubit interaction is always present: imperfect knowledge of the ancilla state during or after readout dephases the qubits^20^,^21^,^22^. To minimize this dephasing, we implement quantum non-demolition measurements of the ancilla by resonant optical excitation of 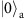 and by stopping the excitation within 2 μs upon photon detection (outcome 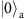) to minimize uncontrolled spin flips in the optically excited state^23^ (Methods). The resulting readout fidelities are *F*_0_=0.890(4) for 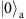 and *F*_1_=0.988(2) for 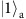 (average: *F*=0.939(2)). Crucially, the post-measurement fidelity after correctly assigning 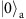 is 0.992, demonstrating the desired non-demolition character.

To benchmark the stabilizer measurements and real-time feedback, we deterministically entangle two qubits by projecting into a Bell state, that is, a simultaneous eigenstate of *XX* and *ZZ*^21^,^24^,^25^. First, the qubits are initialized in 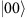, an eigenstate of *ZZ*, with fidelity 0.910(6). Then, a *XX* stabilizer measurement projects the qubits onto one of two Bell states (Fig. 1d). We interpret the −1 outcome as an error in the desired state and correct it through feedback before performing two-qubit tomography. The deterministically generated entangled state, with fidelity *F*=0.824(7) (Fig. 1e), demonstrates the non-destructive nature of the measurement; coherence within the subspaces is maintained throughout the measurement and feedback cycle. The complete cycle can be repeated up to six times within the shortest qubit 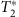.

### Active quantum error correction on a logical qubit

**Figure 2:**
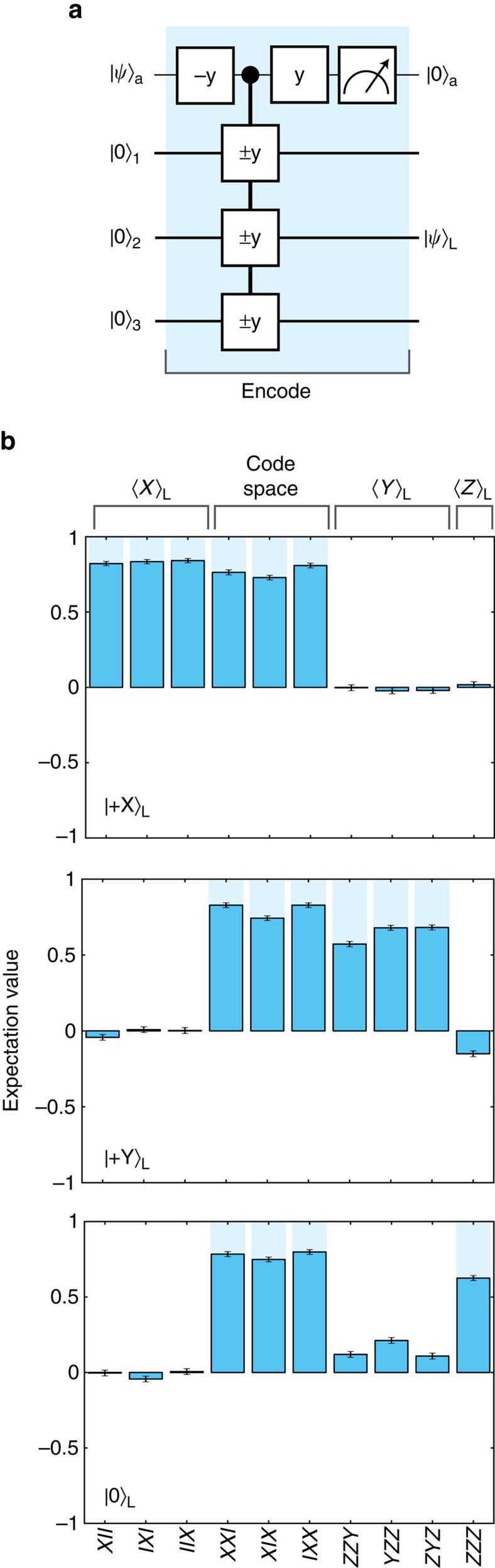
Encoding of the logical qubit. (**a**) Encoding an arbitrary quantum state 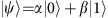 prepared on the ancilla into 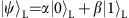. Successful encoding is heralded by outcome 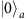. (**b**) Characterization of the logical states 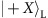, 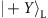 and 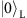. Only the logical qubit operators and stabilizers are shown (see Supplementary Fig. 7 for complete tomography of all 6 logical basis states). The fidelities with the ideal three-qubit states are *F*=0.810(5), 0.759(5)and 0.739(5), respectively, demonstrating three-qubit entanglement^10^. The logical state fidelities are 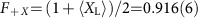, 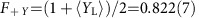 and 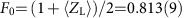. Ideally, all the encoded states are +1 eigenstates of the stabilizers *X*_1_*X*_2_*I*_3_ and *I*_1_*X*_2_*X*_3_. The fidelity to this code space, 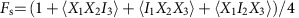, is 0.839(3) averaged over all states and gives the probability that the starting state is free of detectable errors. All error bars are one statistical s.d.

We now turn to quantum error correction by stabilizer measurements. The logical qubit is encoded by mapping an arbitrary state 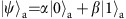 prepared on the ancilla to the three-qubit state 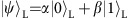 (Fig. 2a). We characterize the encoding by preparing six basis states 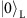, 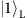, 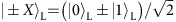 and 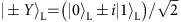 and performing three-qubit state tomography. The fidelities with the ideal states confirm successful encoding and genuine three-qubit entanglement (Fig. 2b).

**Figure 3:**
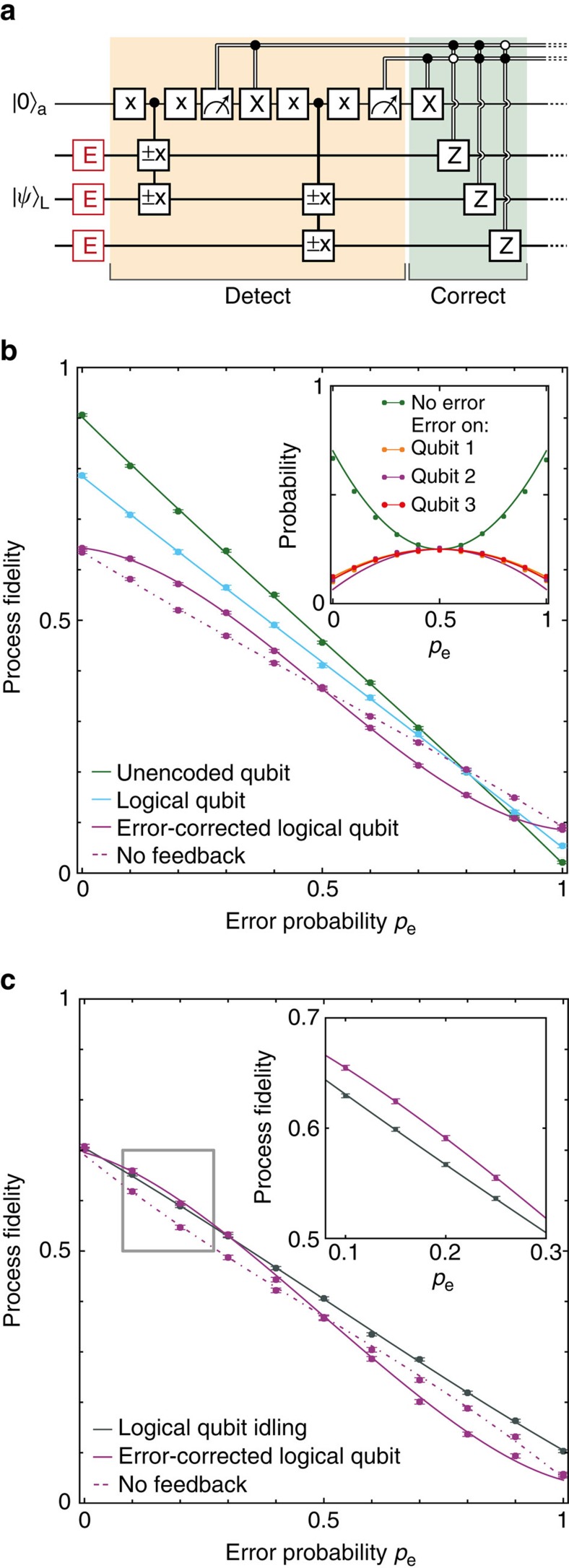
Active quantum error correction by stabilizer measurements. (**a**) All qubits are simultaneously subjected to uncorrelated phase errors *E* with probability *p*_e_. Errors are detected by measuring *X*_1_*X*_2_*I*_3_ and *I*_1_*X*_2_*X*_3_ and subsequently corrected by *Z* operations through feedback. Finally, we measure the process fidelity with the identity. (**b**) Process fidelities for: an unencoded qubit (averaged over the three qubits), the logical qubit without stabilizer measurements, the error-corrected logical qubit and the logical qubit without feedback (that is, errors are detected but not corrected). We average over the logical qubit permutations, for example, *X*_L_=*X*_1_*I*_2_*I*_3_, *I*_1_*X*_2_*I*_3_ and *I*_1_*I*_2_*X*_3_, and the four ways to assign the ancilla states to the error syndromes (see Supplementary Fig. 8 for individual curves). Inset: probabilities for the error syndromes with theoretically predicted curves based on the state tomography in Fig. 2b (Supplementary Note 2). (**c**) Comparison between the error-corrected logical qubit and the logical qubit with the stabilizer measurements replaced by an equivalent idle time (2.99 ms). Compared with **b**, the effective readout fidelity is optimized by associating syndrome +1, +1 (no error) to obtaining 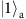 for both stabilizer measurements. Curves in **b**,**c** are fits described in the Methods. All error bars are one statistical s.d.

We first investigate the recovery of arbitrary logical qubit states from phase errors. To emulate a general process causing dephasing, uncorrelated incoherent errors are applied with variable probability *p*_e_ to each physical qubit simultaneously (Fig. 3a); for each qubit, the error process is 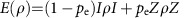, with *ρ* the single-qubit density matrix. By controllably applying such errors, we characterize the effectiveness of the error correction for any process causing uncorrelated errors with equal probability to the qubits. We then measure the stabilizers *X*_1_*X*_2_*I*_3_ and *I*_1_*X*_2_*X*_3_, identify potential errors and correct them through feedback. The probabilities to obtain the four different error syndromes (inset in Fig. 3b) show the expected symmetry around *p*_e_=0.5 and match the theoretical prediction based on the errors present in the initial states (Fig. 2b) and the average ancilla readout fidelity.

The protection of the logical qubit is characterized by the process fidelity with the identity (Fig. 3b; Methods). We quantitatively analyse the results by fitting to 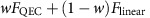, where *F*_QEC_(*p*_e_) and *F*_linear_(*p*_e_) are the theoretical curves with and without error correction (*w*=1 indicates ideal robustness against applied single-qubit errors). When no error correction is applied we observe the expected linear dependence on the error probability: *w*≈0. In contrast, with quantum error correction *w* is 0.81(3), and a nonlinear curve shape that is characteristic for robustness against single-qubit errors is obtained. This result demonstrates that the entropy associated to the applied errors is successfully removed from the system.

Comparisons to an unencoded qubit and the logical qubit without error correction reveal that adding quantum error correction on top of a computation does not yet provide a net improvement (Fig. 3b), because of additional errors introduced by the initialization, encoding and stabilizer measurements (total of 13 two-qubit gates, 488 ancilla refocusing pulses and 6 ancilla readouts/resets). To isolate the errors due to the stabilizer measurements, we compare the error-corrected logical qubit to the logical qubit left idle. We further optimize the error correction, by assigning the ancilla state with the best readout fidelity (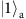, *F*_1_=0.988(2)) to the most likely error syndrome (+1, +1—no error, inset Fig. 3b), instead of averaging over all assignments as in Fig. 3b. With this improvement, error correction outperforms idling for a range of *p*_e_ (Fig. 3c); once the logical qubit is encoded, quantum error correction can be beneficial.

### Multiple rounds of active error correction

**Figure 4:**
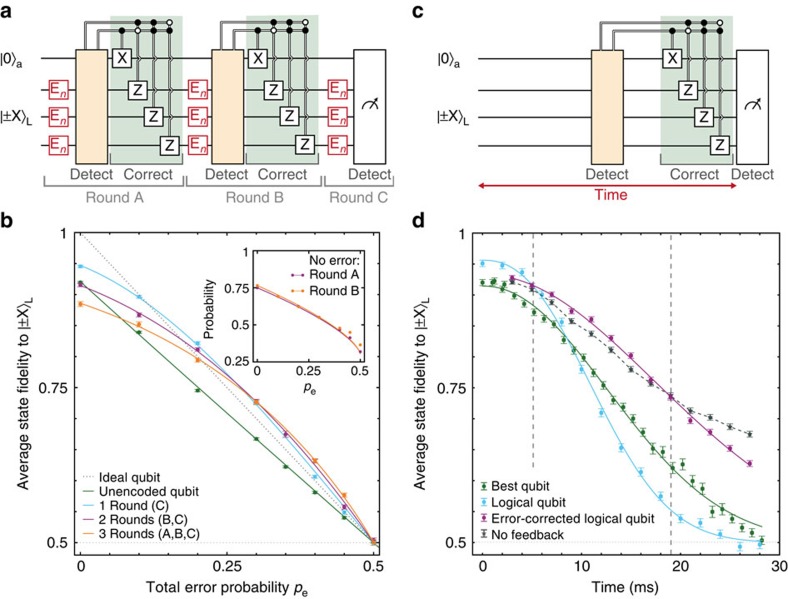
Extending coherence by active quantum error correction. (**a**) Three rounds of error correction on a logical qubit. The first two rounds of quantum error correction use stabilizer measurements and feedback. The final round is implemented by majority voting. (**b**) Average logical state fidelity for 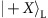
and 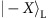 as a function of total error probability *p*_e_ for *n*=1, 2 and 3 rounds of error correction compared with an unencoded qubit. The errors per round *E*_*n*_ occur with probability *p*_*n*_. Inset: probabilities that no error is detected (*n*=3). The similarity of the results for rounds A and B confirms that errors are corrected in between rounds. (**c**) Correcting natural dephasing. The storage time is defined from the end of the encoding until the start of the final measurements. (**d**) Dephasing of the logical qubit: without stabilizer measurements, with quantum error correction and without feedback, compared with the best unencoded qubit. The dashed lines indicate the times between which the actively error-corrected logical qubit gives the highest fidelity. The data without feedback (detecting errors without correcting) isolate the suppression of coherently evolving errors by projecting them. For long times, applying error correction lowers the fidelity because the stabilizer measurements extract no useful information about errors, but nevertheless preferentially suppress evolutions that result in phase errors at the end of the sequence (see Supplementary Fig. 10 for a detailed analysis). See Supplementary Fig. 9 for error syndrome probabilities. Solid curves in **b**,**d** are fits described in the Methods and Supplementary Notes 1 and 2. Dashed lines are a guide to the eye. All error bars are one statistical s.d.

Because a complete round of error correction (2.99 ms) fits well within the dephasing time of the physical qubits, we can concatenate multiple rounds to improve the coherence of continuously encoded quantum superpositions by preventing the accumulation of errors (Fig. 4a). Three new elements are introduced. First, the total error probability *p*_e_ is distributed over *n* rounds, so that the error probability per round is 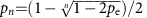 (Methods). This error model corresponds to errors occurring incoherently, for example with a constant rate in time. Second, to investigate dephasing we focus on the protection of the two states 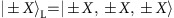 (that is, a classical bit stored in the phase of a quantum superposition). Third, we exploit the intrinsic robustness of the logical qubit to single *Z* errors by redefining 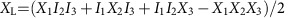, which is equivalent to performing a round of error correction by majority voting at the end of the experiment^13^,^14^.

For a single round of error correction (majority vote only), the average fidelity is higher than for an unencoded qubit for any *p*_e_ (Fig. 4b); adding more (identical) qubits is always beneficial in the repetition code. For *p*_e_=0, additional rounds of quantum error correction can only introduce errors, reducing the fidelity (Fig. 4b). For larger *p*_e_, however, multiple rounds prevent errors from accumulating by dividing the error process in parts that are more likely to contain only single errors, which are corrected. In addition, unlike error detection with post processing^13^,^14^, active correction between rounds keeps the probability to obtain +1 (no error) high (inset Fig. 4b) and thus maintains the advantage of assigning the highest-fidelity ancilla readout to that outcome. Preventing errors by maximizing the probability that the ancilla qubits reside in the optimal state is a key general advantage of real-time feedback in quantum error correction. As a result, for *p*_e_>0.3, multiple rounds outperform a single round of error correction.

### Correcting natural dephasing

Finally, as an example of suppressing errors naturally present in the environment, we let the qubits evolve freely instead of applying errors (Fig. 4c). The resulting errors are still spatially uncorrelated across the qubits, but the error probabilities are now different for each qubit because their intrinsic 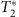 differ because of their local environments (Fig. 1c). In addition, the errors arise from quasistatic detunings because of the slowly fluctuating ^13^C spin bath so that the errors in a given experimental run evolve coherently and are correlated in time. Like most environmental errors, such errors might also be suppressed by other methods than quantum error correction, for example, by polarizing the spin environment^26^,^27^, by refocusing pulses^28^ or by isotopic purification^28^,^29^,^30^,^31^.

The fidelity for the logical qubit with majority voting again starts above the best unencoded qubit, but drops below it for larger evolution times (Fig. 4d). Because the error probabilities vary between qubits, an error detected on the best qubit becomes more likely to actually correspond to errors on both other qubits and the wrong correction is made. An additional round of quantum error correction in the middle of the evolution time now not only prevents errors from accumulating by intermediately correcting them, but also interrupts any coherent build-up by projecting the errors, thus suppressing them (Fig. 4d). Owing to this combination, the logical qubit shows an enhanced dephasing time (24.2(2) ms against 18.2(9) ms for the best physical qubit) and yields the highest average state fidelity for total evolution times between 5 and 19 ms (Fig. 4d). This result demonstrates an actively error-corrected logical qubit with an improved dephasing time over the best qubit used in the encoding.

## Discussion

The presented non-destructive measurements and real-time feedback on encoded quantum states are the key primitives for universal computations on logical qubits and for error-correcting codes that correct both phase and bit-flip errors. To reach scalability thresholds, readout and gate fidelities should be further increased, for example, by: improving the optical collection efficiency through optical cavities^32^, enhancing coherence times through implantation^33^ or selective growth of defects and isotopes in purified diamonds^28^,^29^, and improving gate design through optimal control^33^. In a wider perspective, our results can be combined with recently demonstrated entanglement between distant NV centres^34^,^35^ to form quantum networks with error-corrected nodes for entanglement purification, quantum communication and networked quantum computation^36^. Therefore, these results establish a promising platform to experimentally investigate protocols for fault-tolerant quantum information processing under different types of noise and error correlations in diverse settings.

## Methods

### Sample and setup

We use a naturally occurring NV in high-purity type IIa chemical-vapour-deposition-grown diamond with a 1.1% natural abundance of ^13^C and a <111> crystal orientation (Element Six). To enhance the collection efficiency, a solid-immersion lens was fabricated on top of the NV centre^17^,^37^ (Fig. 1b) and a single-layer aluminum-oxide anti-reflection coating was deposited^34^,^38^. The sample temperature is *T*≈4.2 K and a magnetic field of 403.553(3) G is applied along the NV symmetry axis.

The ancilla NV electron spin is characterized by a Rabi frequency of 4.3 MHz, a dephasing time 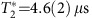, a Hahn echo time *T*_2_=1.03(3) ms and a longitudinal relaxation time of 0.43(6) s (due to microwave noise and laser background). The coherence time of the ancilla under dynamical decoupling exceeds 25 ms and does not limit the experiments (Supplementary Fig. 1). We initialize and readout the ancilla through resonant excitation of the zero-phonon transitions of the NV centre (Supplementary Fig. 2). Before every experiment, the ^14^N nuclear spin is initialized by measurement with a fidelity of *F*_N_=0.94(3) in *m*_I_=−1 (ref. 17). No external electric fields are applied: the gates in Fig. 1b are grounded.

### Nuclear spin qubit control

The hyperfine interactions for the three nuclear spins are estimated by dynamical decoupling spectroscopy^9^ (Supplementary Table 1). Building on previous gate designs^9^, nuclear gates are realized by applying sequences of π-pulses on the electron spin of the form (*τ*−*π*−2*τ−π−τ*)^*N*/2^. The number of pulses *N* sets the rotation angle. The inter-pulse delay 2*τ* determines which qubit is controlled and whether the rotation is conditional on the ancilla state. In contrast to the previous work^9^, we allow the gates to be detuned, providing greater flexibility to optimize *τ* and *N* for gate selectivity and minimal discretization errors. The gate parameters are listed in Supplementary Tables 1 and 2.

The nuclear spins are initialized by swapping with the ancilla electron spin (Supplementary Fig. 3) and are read out by mapping the required correlation to the ancilla before reading it out (Supplementary Fig. 4). To obtain best estimates for the actual states, the results are corrected for the fidelity of the gates used in the final readout (tomography; details in Supplementary Note 3). Uncorrected data are shown in Supplementary Fig. 11.

### Feedback

Real-time feedback is implemented through a programmable microprocessor (ADwin Pro II) that controls the experimental sequence (Supplementary Fig. 5). We exploit feedback in four different ways. First, detected phase errors are corrected directly after the stabilizer measurements. Note that analysing errors over multiple rounds^14^ would additionally enable real-time correction of ancilla readout errors, but that this is not implemented here. Second, depending the ancilla measurement outcome, the qubits pick up a deterministic phase shift due to the hyperfine interaction, which is corrected in the same way. Third, for an odd number of +1 outcomes, the operations in the stabilizer measurements imprint a bit flip on the logical qubit, which we correct by transforming the logical qubit basis in real time. Fourth, to start each measurement sequence with the ancilla in 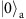, it is flipped back to 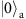 when the previous measurement returned 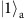.

Importantly, we perform real-time feedback either by adapting the qubit bases for all subsequent gates and measurements (for correcting *Z* errors and for the logical qubit) or by absorbing the feedback operations into the next gate acting on the same qubit (for the ancilla). Therefore, the physical control sequence is directly adapted based on the measurement outcomes without introducing any unnecessary gate operations that would cause additional errors. In the circuit diagrams, we sometimes display the gates for the feedback separately for clarity.

### Quantum error correction analysis

The process fidelity with the identity is given by 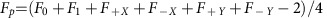, with 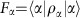, the six fidelities of the final states *ρ*_*α*_ with the ideal states 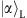. The results of Fig. 3 are analysed by fitting to 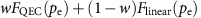, with 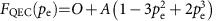 and 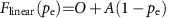. *A* and *O* account for the experimental fidelities (Supplementary Note 1).

The state fidelities for multiple rounds of error correction and incoherent errors (Fig. 4b) are fitted to the same equation using 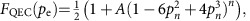 with *n* the number of rounds, *p*_*n*_ the error per round and 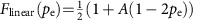. The error per round *p*_*n*_ is obtained as follows. An error process with total error probability (*p*_e_) reduces the expectation value by a factor of (1−2*p*_e_). For incoherent errors, a process can be divided in *n* equal rounds using (1−2*p*_e_)=(1−2*p*_*n*_)^*n*^, which results in 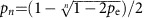 (for *p*_e_≤0.5). In Figs 3c and 4b, *A* depends on the error-probability *p*_e_, because we optimize the effective readout fidelity by associating the most likely error syndrome to the best ancilla readout (Supplementary Note 1). See Supplementary Notes 1 and 2 for further details on all theoretical analysis, including the error syndrome probabilities and numerical simulations of Fig. 4d.

## Additional information

**How to cite this article:** Cramer, J. *et al*. Repeated quantum error correction on a continuously encoded qubit by real-time feedback. *Nat. Commun.* 7:11526 doi: 10.1038/ncomms11526 (2016).

## Supplementary Material

Supplementary Information Supplementary Figures 1-11, Supplementary Tables 1-2, Supplementary Notes 1-3 and Supplementary References
