# Supplementary material for: Repeated quantum error correction on a continuously encoded qubit by real-time feedback
Source: Nat Commun. 2016 May 5;7:11526. doi: 10.1038/ncomms11526 (PMC4858808; doi:10.1038/ncomms11526)
Supplement: Supplementary Information — Supplementary Figures 1-11, Supplementary Tables 1-2, Supplementary Notes 1-3 and Supplementary References [file ncomms11526-s1.pdf]

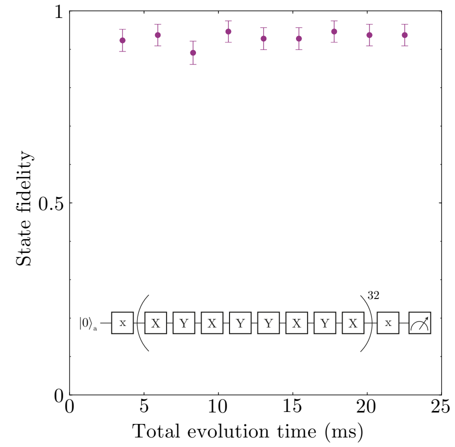

Supplementary Figure 1: Coherence of the NV electron spin by dynamical decoupling with 256 pulses. The spin is decoupled from the nuclear spin bath by applying a sequence of 256 π-pulses with alternating phases. The time between the pulses is chosen to be a multiple of the Larmor period of the ^{13}C spins. This result shows no significant decay on the relevant timescale of our experiments. Error bars are one statistical s.d.

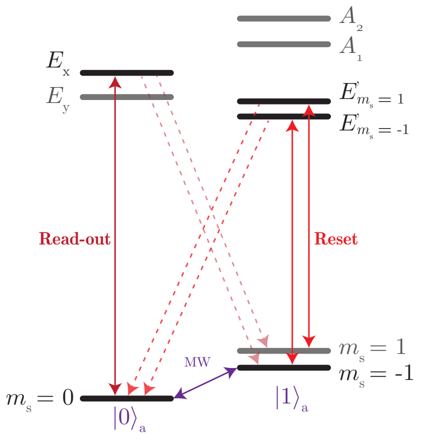

Supplementary Figure 2: Optical readout and initialization of the ancilla NV electron spin. The electron spin is initialized and read out by spin-selective resonant excitation [2]. To initialize or reset the electron spin state we apply a laser pulse that excites only the $m_s = \pm 1 \leftrightarrow E'_{m_s=\pm 1}$ transitions (Reset). Due to spin mixing in the excited state this prepares the electron spin in the $m_s = 0$ state (fidelity > 0.98) [2]. To measure the spin state we apply a laser pulse resonant with the $m_s = 0 \leftrightarrow E_x$ transition (Readout). Ideally, this results in the detection of 1 or more photons for the $m_s = 0$ state, and no detected photons for $m_s = \pm 1$. The resulting readout fidelities are asymmetric: $F_0 = 0.890(4)$ for $m_s = 0$ (limited by the detection efficiency and number of cycles before a spin flip) and $F_1 = 0.988(2)$ for $m_s = \pm 1$ (limited by background counts and unwanted excitations). Because uncontrolled spin flips in the excited state decohere nearby nuclear spins, we minimize the number of unnecessary optical excitations by using a weak readout pulse with a maximum duration of 114 μs ($\sim$ 100 excitations) and by switching off the laser within 2 μs ($\sim$ 2 excitations) once a photon is detected [3]. The resulting measurement is non-destructive: the probability that the spin prepared in $m_s = 0$ is still in that state after a measurement with outcome $m_s = 0$ is 0.992. In contrast, without dynamically stopping the laser the spin would be pumped almost completely to $m_s = \pm 1$. For the final readout at the end of the experiment, which is allowed to be destructive, we use a stronger readout pulse of maximum duration 35 μs.

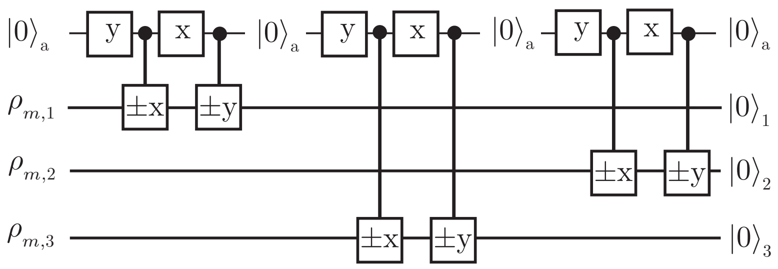

Supplementary Figure 3: Qubit initialization. All qubits naturally start in fully mixed states ρ_{m}. The ancilla is initialized in $|0\rangle_{\mathrm{a}}$ and a reduced SWAP operation between the ancilla and the qubit is performed, deterministically initializing the qubit in $|0\rangle$. The ancilla is then reinitialized by a 300 μs laser pulse (Reset) and the process is repeated to initialize the other qubits.

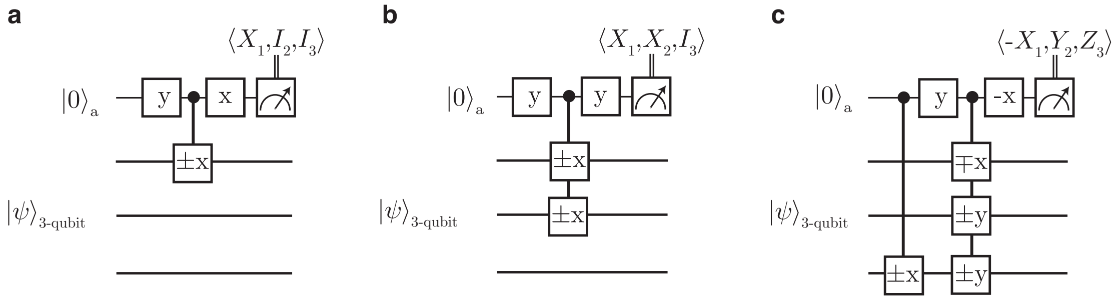

Supplementary Figure 4: Tomography sequences for three-qubit states. Examples of three-qubit expectation values that are measured by mapping the required correlation on the ancilla before reading it out. **a,** $\langle X_1, I_2, I_3\rangle$, **b,** $\langle X_1, X_2, I_3\rangle$, **c,** $\langle -X_1, Y_2, Z_3\rangle$. Note that the phase of the last $\pi/2$-pulse on the ancilla depends on the number of qubits read out (i.e. the number of operators that are not I). The examples given here for the measurement of one- two- and three-qubit expectation values can be translated to any of the 63 measurements in the full three-qubit state tomography.

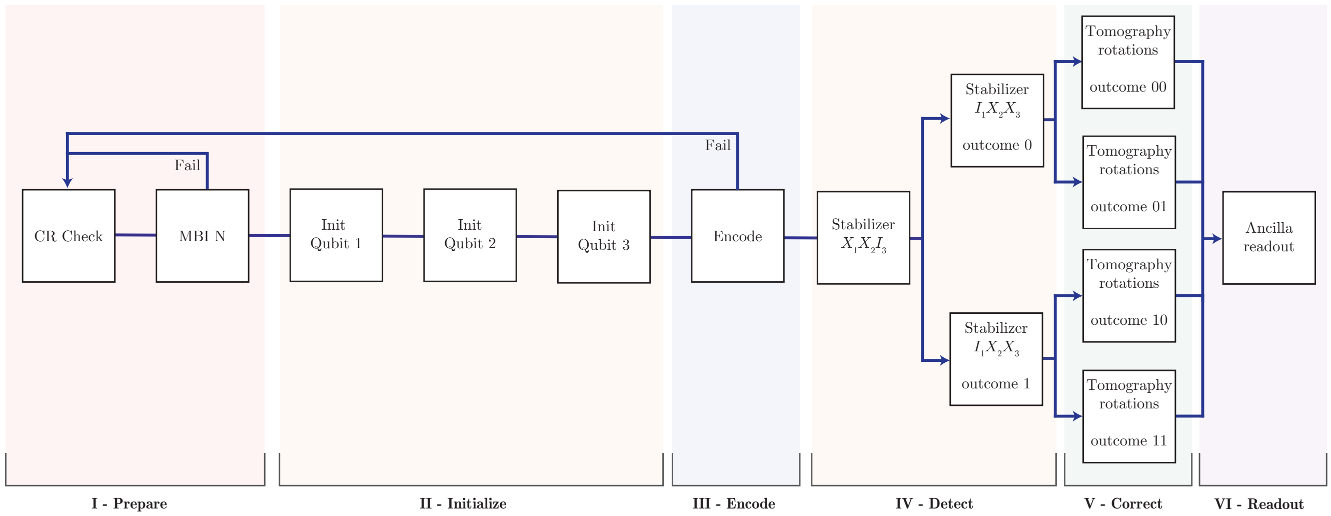

Supplementary Figure 5: Experimental sequence and logic for the QEC experiments. Example for a single round of quantum error correction by stabilizer measurements (as in Fig. 3). The order of the sequence is controlled in real-time by an ADwin microprocessor. **I -** The NV center is prepared in its negative charge state and on resonance with the readout and reset lasers (Supplementary Fig. 2) by turning on both lasers, counting the fluorescence photons and requiring a threshold to be passed ("CR Check"). The ^{14}N nuclear spin is initialized by measuring it and continuing only for outcome $m_{\mathrm{I}} = -1$ ("MBI N", success probability 0.073(7)). **II -** The qubits are sequentially deterministically initialized following Supplementary Fig. 3. **III -** The encoding of the logic state is a probabilistic process (success probability 0.41(1)), as shown in Fig. 2a. When the wrong outcome is obtained the preparation of the experiment starts over. **IV -** Errors are detected by two stabilizer measurements. Depending on the outcome (-1 or $+1$) of each measurement, the next sequence to execute is communicated to the waveform generator in real time. **V -** Depending on which of the 4 outcomes is obtained, a set of gates is performed to correct errors and to map the desired expectation value onto the ancilla (Supplementary Fig. 4). **VI -** Finally the ancilla is read out. Each outcome is taken into account without post processing or post selection. Note that for the experiment with three rounds of error correction (two rounds of stabilizer measurements QEC, Fig. 4b), the sequence branches in 16 paths instead.

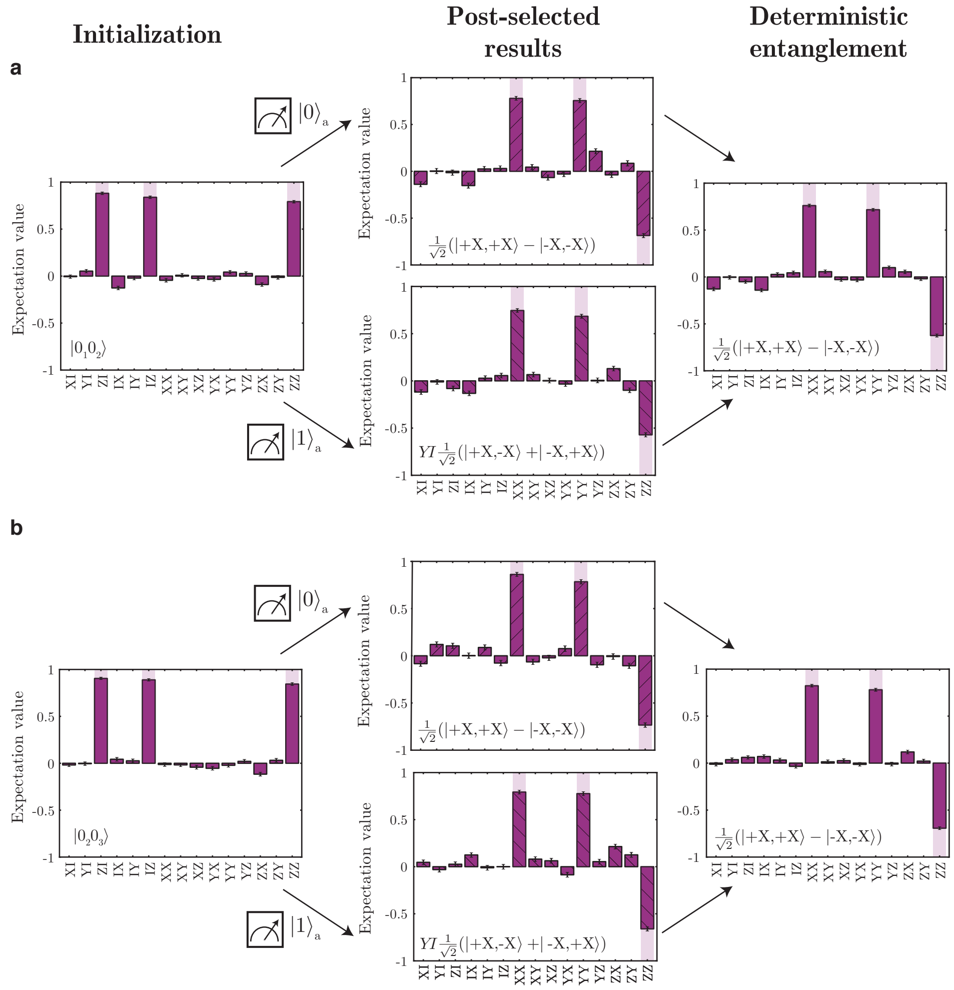

Supplementary Figure 6: Deterministic entanglement by stabilizer measurements including post-selected results. a, For qubits 1 and 2. **b,** For qubits 2 and 3. First the qubits are initialized following Supplementary Fig. 3 in $|00\rangle$ with fidelity 0.878(6) for (a) and 0.910(6) for (b) (left column). Then a XX measurement is performed (Fig. 1d). Depending on the measurement outcome feedback is applied, so that independent of the outcome the same two-qubit state is obtained, as can be seen by post-selecting on the two outcomes (middle column). The full result is a deterministically entangled state (right column). The fidelity with the desired two-qubit entangled state is 0.776(7) in (a) and 0.824(7) in (b).All error bars are one statistical s.d.

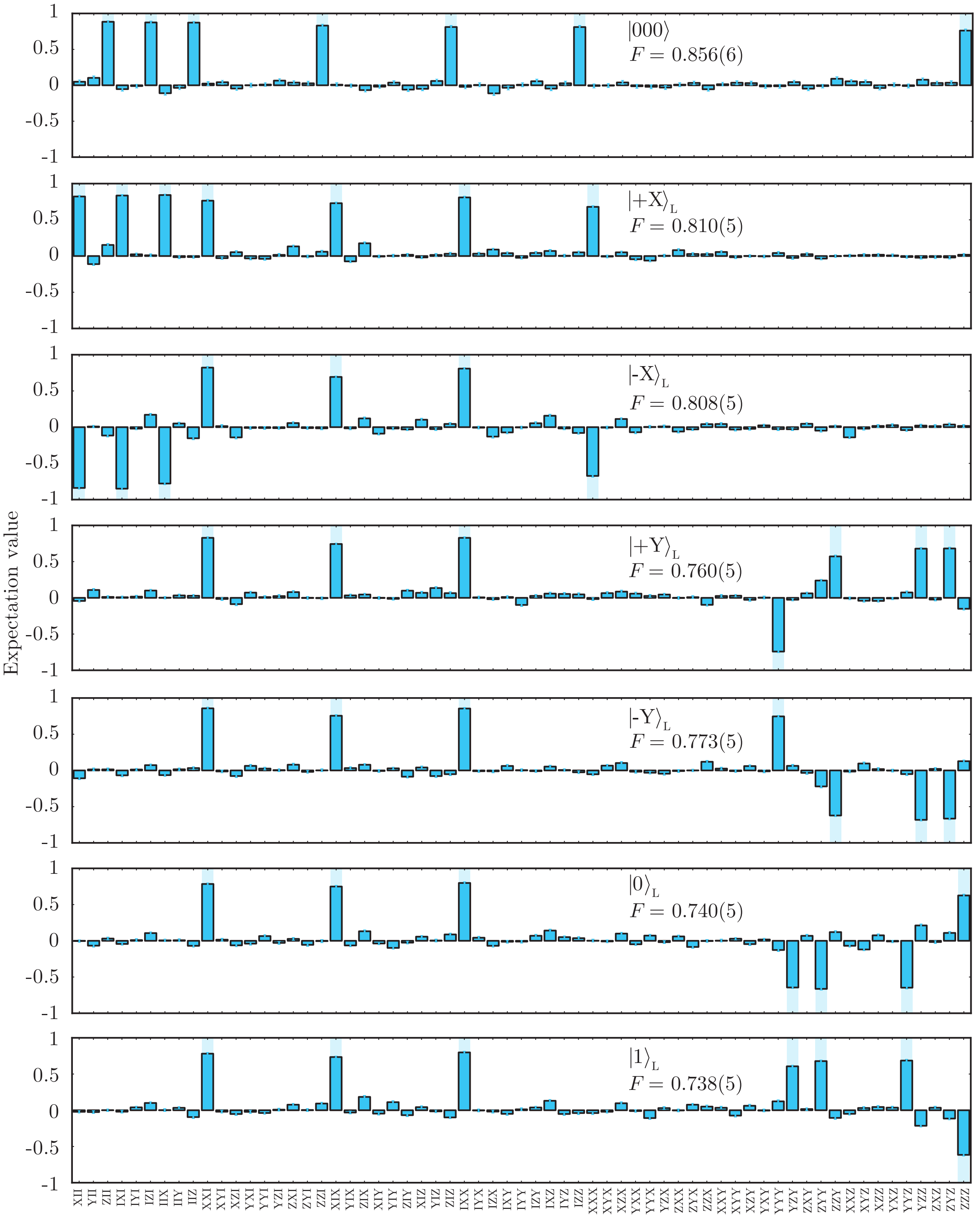

Supplementary Figure 7: Three-qubit state tomography for $|000\rangle$ and the six logical states. The three qubits are initialized as in Supplementary Fig. 3 and encoded following Fig. 2a. The fidelities F shown are the three-qubit state fidelities and the shaded bars indicate the ideal states. Ideally, the 6 encoded states are all eigenstates of the XX stabilizers with eigenvalues +1, in agreement with the high values for $\langle X_1, X_2, I_3\rangle$, $\langle X_1, I_2, X_3\rangle$ and $\langle I_1, X_2, X_3\rangle$ for all states and an average fidelity with this code subspace of 0.839(3). The logical qubit is encoded as $\alpha|0\rangle_\mathrm{L} + \beta|1\rangle_\mathrm{L}$, with $|0\rangle_\mathrm{L} = \frac{1}{\sqrt{2}}(|\text{+X,+X,+X}\rangle + |\text{-X,-X,-X}\rangle)$ and $|1\rangle_\mathrm{L} = \frac{1}{\sqrt{2}}(|\text{+X,+X,+X}\rangle - |\text{-X,-X,-X}\rangle)$. The logical state expectation values are given by: $\langle X\rangle_\mathrm{L} = \langle X_1, I_2, I_3\rangle$, $\langle Y\rangle_\mathrm{L} = \langle Y_1, Z_2, Z_3\rangle$, $\langle Z\rangle_\mathrm{L} = \langle Z_1, Z_2, Z_3\rangle$ or cyclic permutations. The logical qubit fidelities for the states are $|+X\rangle_\mathrm{L}: 0.916(6)$, $|-X\rangle_\mathrm{L}: 0.911(6)$, $|+Y\rangle_\mathrm{L}: 0.822(7)$, $|-Y\rangle_\mathrm{L}: 0.828(7)$, $|0\rangle_\mathrm{L}: 0.813(9)$ and $|1\rangle_\mathrm{L}: 0.808(9)$. All error bars are one statistical s.d.

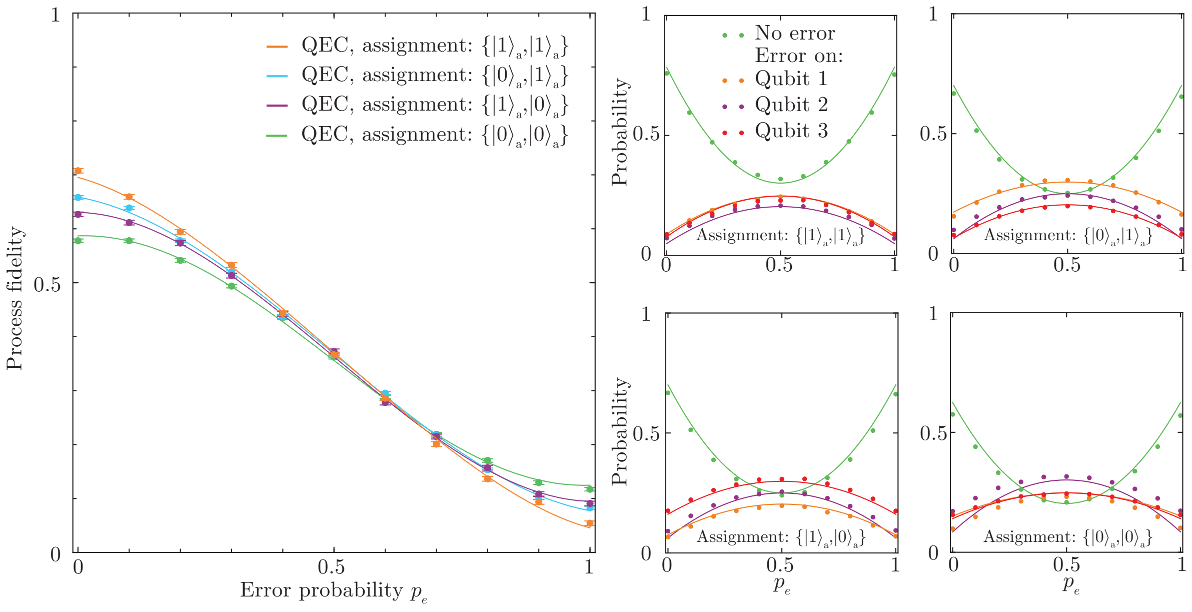

Supplementary Figure 8: Process fidelity and error syndrome probabilities for different ancilla assignments. Left: comparison of the process fidelities for the quantum error correction experiment in Fig. 3 for all four possible assignments of the ancilla states to the +1,+1 outcome of the stabilizer measurements. In Fig. 3b we average over these four curves. In Fig. 3c the optimal result is used (assignment $\{|1\rangle_a, |1\rangle_a\}$). Solid lines are fits to Eq. 3 taking into account Eq. 4 and yield: $w = 0.8(1)$, $w = 0.71(7)$, $w = 0.95(7)$ and $w = 0.84(9)$ for the four assignments. Right: the probabilities for the error syndromes for each of the four ancilla state assignments. Solid lines are expected curves similar to Eqs. 24-27, based on the estimated initial errors in the encoded states: $p_{\mathrm{in}}^{(1)} = 0.091(2)$, $p_{\mathrm{in}}^{(2)} = 0.064(2)$, $p_{\mathrm{in}}^{(3)} = 0.077(2)$ obtained from Fig. 7. The theoretical probabilities are in good agreement with the experimental values (no free parameters). The probabilities are the normalized occurrences in 84000 samples for the assignments $\{|0\rangle_a, |0\rangle_a\}$ and $\{|0\rangle_a, |1\rangle_a\}$ and in 28000 samples for the assignments $\{|1\rangle_a, |1\rangle_a\}$ and $\{|1\rangle_a, |0\rangle_a\}$. All error bars are one statistical s.d.

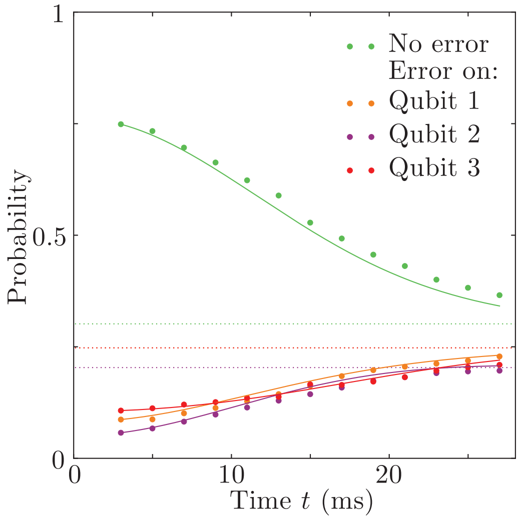

Supplementary Figure 9: Error syndrome probabilities for naturally occurring errors. Corresponding to Fig. 4d. Solid lines are theoretical predictions from the unique coherence times T_2^* of the individual qubits and the initial error (p_{in}) determined from this data. As the stabilizer measurements are performed halfway the waiting time, the error probability for each qubit is: $p_e(\frac{t}{2}) = \frac{1}{2}(1 - \mathrm{Exp}[-(\frac{t}{2T_2^*})^2])$. Using Eqs. 24-27 and the measured error outcome probabilities at the first datapoint ($t = 2.99$ ms), we estimate the input errors at $t = 0$ to be $p_{\mathrm{in}}^{(1)} = 0.049(2)$, $p_{\mathrm{in}}^{(2)} = 0.0804(4)$ and $p_{\mathrm{in}}^{(3)} = 0.110(2)$. Dashed lines show the expected probabilities for complete dephasing. The probabilities are based on the normalized occurrences in 12000 samples.

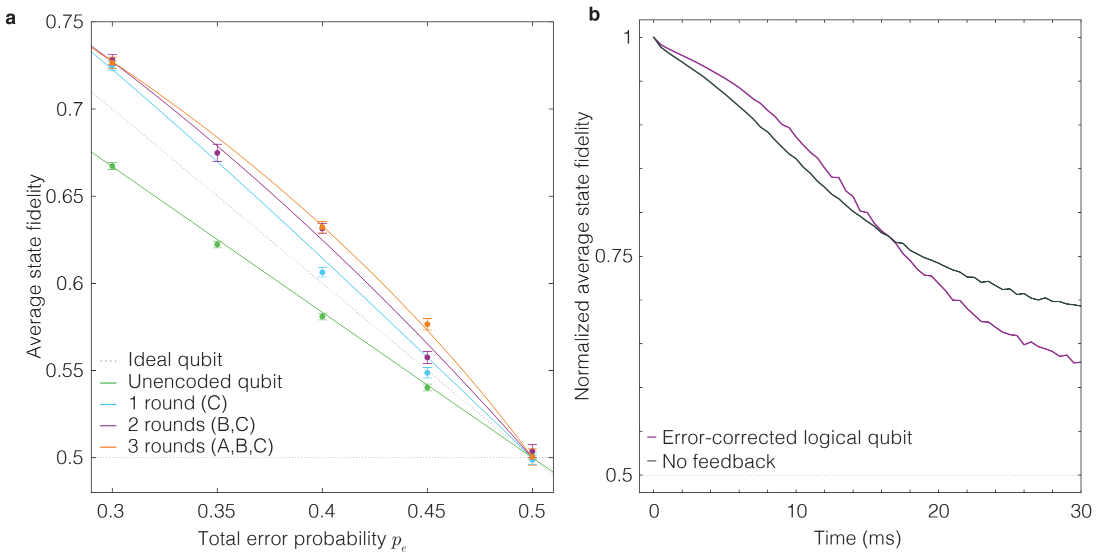

Supplementary Figure 10: Zoom of data of Fig. 4b (a) and numerical simulation of Fig 4d (b). a, A zoom-in of the area of the data in Fig. 4b in which additional rounds of error-correction are advantageous. **b,** Numerical Monte-Carlo simulations for the error correction experiment of Fig 4d. **Sequence.** The initial state is $|X\rangle_{\mathrm{L}}$. Each qubit then coherently evolves with a constant detuning randomly drawn from a Gaussian probability distribution with $\sigma = \sqrt{2}/T_2^*$ for that qubit. Halfway the evolution time the stabilizer measurements instantaneously project the quantum state, taking into account the asymmetric fidelity of the ancilla readout and the error-dependent readout fidelity (Eqs. 3-8). After letting the state evolve for the second period with the same detuning, detected errors are corrected (this final step is omitted for "No feedback"). **Longitudinal relaxation.** The qualitative behavior of the simulations is dominated by the dephasing times T_2^*. We additionally take into account the measured longitudinal relaxation of each qubit, which approximately decays with $e^{-(t/T_1)^{0.5}}$ for ancilla state $|0\rangle_{\mathrm{a}}$ (See Tab. I), and of the ancilla (time-constant 300 ms, due to MW and laser background). The longitudinal decay results in a small quantitative correction, but does not alter the qualitative behavior observed. **Results & discussion.** The simulation results qualitatively match all the main features of the observed dephasing curves (Fig. 4d). For short times, the stabilizer measurements suppress errors by stopping small errors from building up coherently and error correction further reduces the remaining errors. For long times, the stabilizer measurements halfway the sequence preferentially suppress coherent evolutions that would result in an error at the end of the sequence. As a result the fidelity at long times exceeds 0.5 and decays only slowly. Moreover, for long times, applying error correction becomes detrimental: at the moment the stabilizer measurements are applied the state is essentially random and no useful information about errors is extracted so that applied corrections further dephase the final state. A complete quantitative comparison would require detailed modeling of the full evolution of the 4-qubit system during the gates, initialization, stabilizer measurements, and readout sequences as well as of the longitudinal decay at short times. All error bars are one statistical s.d.

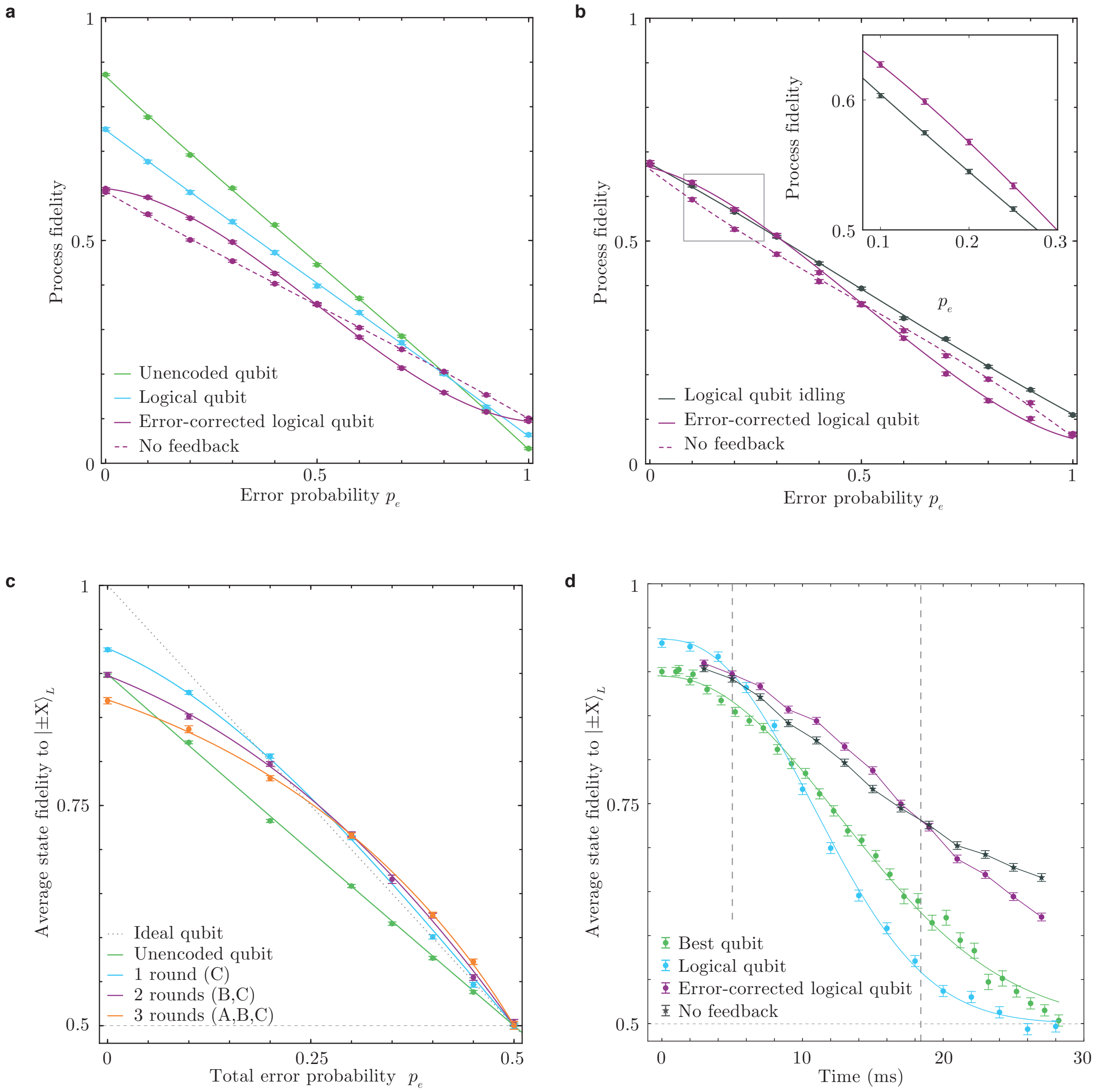

Supplementary Figure 11: Data of Fig. 3b (a) 3c (b), 4b (c) and 4d (d), without correction for the final readout gates. a, b, The fitted value for w is identical as in the main text (curve shapes are not influenced by the readout calibration). **c,** Corresponding fit values for the unencoded qubit: $w = -0.02(2)$, for 1 round: $w = 0.56(6)$, for 2 rounds $w = 0.64(4)$ and for three rounds $w = 0.70(2)$. **d,** The logical qubit follows Eq. 12 with $T = 13.7(1)$ ms and $n = 2.35(8)$. All error bars are one statistical s.d.

SUPPLEMENTARY TABLES

	Qubit 1	Qubit 2	Qubit 3
$A_{\parallel}$ (kHz)	$2\pi\cdot 20.6$	$2\pi\cdot$-36.4	$2\pi\cdot 24.4$
$A_{\perp}$ (kHz)	$2\pi\cdot 43$	$2\pi\cdot 25$	$2\pi\cdot 26$
ω_0 (kHz)	$2\pi\cdot 431.874(3)$	$2\pi\cdot 431.994(3)$	$2\pi\cdot 431.934(3)$
ω_1 (kHz)	$2\pi\cdot 413.430(3)$	$2\pi\cdot 469.025(3)$	$2\pi\cdot 408.303(3)$
τ (μs)	13.616	4.996	11.312
N	32	34	48
gate time (μs)	980	400	1086
T_2^*, $m_s = \ \ 0$ (ms)	12.0(9)	9.1(6)	18.2(9)
T_2^*, $m_s = -1$ (ms)	12.8(6)	9.8(4)	21(1)
T_1, $m_s = \ \ 0$ (ms)	110(10)	100(10)	330(30)

Supplementary Table I: Qubit and gate parameters. $A_{\parallel}$ and $A_{\perp}$ are the estimated hyperfine interaction components parallel and perpendicular to the applied magnetic field. ω_0 and ω_1 are the nuclear precession frequencies for $m_s = 0$ ($|0\rangle_a$) and $m_s = -1$ ($|1\rangle_a$). τ is half the inter pulse delay, N the number of pulses and *gate time* the total duration for the conditional $\pm x$-gates. These values vary slightly over the experiment as they are calibrated every ~ 36 hours. T_2^* is the (natural) dephasing time and T_1 the longitudinal relaxation time.

	Fig. 3b (logical qubit with QEC)	Fig. 4b (three rounds)
Two-qubit gates	19	20
Ancilla refocusing pulses	698	808
Ancilla read-out and reset	7	9

Supplementary Table II: Experimental complexity. Number of operations in the entire sequence, starting from the initialization of the nuclear spins as qubits. All qubit (^{13}C) gates are composed of ancilla (NV electron spin) refocusing pulses and the ancilla is read-out and reset multiple times. We give values for two examples: a single round of QEC with measurement of $\langle Z_1 Z_2 Z_3\rangle$ (Fig. 3b) and three rounds of QEC with measurement of $\langle X_1 X_2 X_3\rangle$ (Fig. 4b).

SUPPLEMENTARY NOTES

Supplementary Note 1: Theoretical analysis: state and process fidelities

For ideal error correction the process fidelity to the identity as a function of error probability p_e for a single round of quantum error correction (QEC) is

$$F_{\mathrm{QEC}}(p_e) = O + A(1 - 3p_e^2 + 2p_e^3). \tag{1}$$

The offset O and amplitude A account for the finite experimental state fidelities. Note that the value at $p_e = 0.5$, $F_{\mathrm{QEC}}(p_e = 0.5) = O + A/2$, is determined by the fidelity of the logical states $|0\rangle_L$ and $|1\rangle_L$, which are insensitive to phase errors. Without error correction a linear function

(2)

is expected. The experimental data can be fitted to a weighted sum of the two functions by:

(3)

The shape of the curve is set by w, which gives the relative weights of the equations for ideal error correction and for no error correction.

Assignment of ancilla states to the error syndrome: effective measurement fidelity F_M

In our experiment, the ancilla readout fidelity is asymmetric: $|1\rangle_a$ has a higher readout fidelity ($F_1 = 0.988(2)$) than $|0\rangle_a$ ($F_0 = 0.890(4)$). The effective measurement fidelity for error correction F_M is therefore determined by the

probabilities to obtain $|0\rangle_\mathrm{a}$ or $|1\rangle_\mathrm{a}$, which depend on the assignment of the ancilla states ($|0\rangle_\mathrm{a}$ or $|1\rangle_\mathrm{a}$) to each stabilizer measurement outcome (+1 or -1) and the probabilities for different errors to occur. There are four different ways to assign the ancilla states to the error syndromes: the +1,+1 outcome (no error) can be set to result in $\{|0\rangle_\mathrm{a}, |0\rangle_\mathrm{a}\}$, $\{|0\rangle_\mathrm{a}, |1\rangle_\mathrm{a}\}$, $\{|1\rangle_\mathrm{a}, |0\rangle_\mathrm{a}\}$ or $\{|1\rangle_\mathrm{a}, |1\rangle_\mathrm{a}\}$. The probability to obtain outcome +1,+1 (no error) ideally is $1 - 3p_e + 3p_e^2$, while the probability to detect an error on a given qubit is $p_e - p_e^2$. With these probabilities, we obtain the effective QEC measurement fidelity as function of error probability:

$$F_\mathrm{M} = F^{(0)}(1 - 3p_e + 3p_e^3) + (F^{(1)} + F^{(2)} + F^{(3)})(p_e - p_e^2) = F^{(0)} + (F^{(1)} + F^{(2)} + F^{(3)} - 3F^{(0)})(p_e - p_e^2), \quad (4)$$

with $F^{(0)}$, $F^{(1)}$, $F^{(2)}$ and $F^{(3)}$, the readout fidelities for 0 errors, an error on qubit 1, an error on qubit 2 and an error on qubit 3, respectively. For example, for assignment $\{|1\rangle_\mathrm{a}, |1\rangle_\mathrm{a}\}$ to stabilizer outcomes +1,+1 (no error), these readout fidelities are

$$F^{(0)} = F_1^2, \quad (5)$$

$$F^{(1)} = F^{(3)} = F_1 F_0, \quad (6)$$

$$F^{(2)} = F_0^2, \quad (7)$$

In a similar way, the fidelities for the other three assignments can be calculated.

Finally, if we assume that an erroneous ancilla readout decoheres the logical state, the dependence of the effective readout fidelity on p_e can be taken into account by setting:

$$A = A' F_\mathrm{M} \quad (8)$$

in Eqs. 1&2 for the process fidelity, with A' a constant.

Fitting of Figs. 3b, 3c and 8

In Fig. 3b, the ancilla readout is symmetrized by averaging over all four assignments, so that F_M equals the average readout fidelity 0.939(2) and is independent of p_e. We can therefore simply fit the data in Fig. 3b to Eq. 3, with A constant. We find $w = 0.81(3)$, corresponding to an average probability to successfully correct single-qubit errors of $\langle P_n \rangle = \frac{1}{3}(w + 2) = 0.94(1)$ [1]. We obtain $A = 0.557(2)$ and $O = 0.086(1)$. For the unencoded qubit, the encoded qubit without stabilizer measurements, and the encoded qubit without feedback, we find a linear function and $w \approx 0$ ($\langle P_n \rangle \approx 2/3$) as expected without error correction (exact values: $w = -0.06(3), -0.03(3)$ and $-0.07(3)$, $A = 0.882(4), 0.734(3)$ and $0.543(2)$, and $O = 0.019(3), 0.051(3)$ and $0.092(1)$, respectively for the three cases). In Supplementary Fig. 8 the separate process fidelities for the different assignments are shown. Switching between assignments is done by adding or omitting a π-pulse before the ancilla readout.

In Fig. 3c, we assign the ancilla state $|1\rangle_\mathrm{a}$ to the +1 outcome for all stabilizer measurements. This assignment is optimal because it associates the best readout fidelity with the most likely outcome: +1,+1 (no error, inset in Fig. 3b). We fit the data in Fig. 3c and Supplementary Fig. 8 to Eq. 3, with A now error-dependent according to Eq. 8 and obtain $w = 0.8(1)$, corresponding to $\langle P_n \rangle = 0.93(3)$ ($A' = 0.666(8)$ and $O = 0.038(6)$). The values for w and $\langle P_n \rangle$ are in good agreement with the result of Fig. 3b, indicating that the treatment in Eqs. 4&8 is accurate.

Multiple rounds of error correction (incoherent errors), Fig. 4b

For multiple rounds of QEC with incoherent errors and with the total error with probability p_e equally distributed over n rounds, the error-probability per round is $p_n = \frac{1}{2}(1 - \sqrt[n]{1 - 2p_e})$, for $p_e < 0.5$. Ideally, the (average) state fidelity is then described by:

$$F = \frac{1}{2}[1 + (1 - 6p_n^2 + 4p_n^3)^n]. \quad (9)$$

As before we fit the data to a weighted sum of the equations for ideal error correction and for a linear error-dependence (no error correction). We use the optimal ancilla state assignment ($F_\mathrm{M}(p_e)$ from Eq. 4). For two rounds of error correction we obtain

$$F_2 = \frac{1}{2} w[1 + A' F_\mathrm{M}(1 - 6p_2^2 + 4p_2^3)^2] + \frac{1}{2}(1 - w)[1 + A' F_\mathrm{M}(1 - 2p_e)], \quad (10)$$

giving $w = 0.66(4)$ and $A' = 0.850(9)$. For three rounds it becomes

$$F_3 = \frac{1}{2}w[1 + A'F_{\mathrm{M}}^2(1 - 6p_3^2 + 4p_3^3)^3] + \frac{1}{2}(1 - w)[1 + A'F_{\mathrm{M}}^2(1 - 2p_e)], \tag{11}$$

giving $w = 0.71(2)$ and $A' = 0.810(5)$. Importantly, the data for multiple rounds cannot be accurately described by the expected shape for a single round of error correction (Eq. 3).

Naturally occurring decoherence (coherent errors), Fig. 4d

The experiments for the best unencoded qubit, the logical qubit with QEC and without QEC (majority vote only) are fitted to a general exponentially decaying function:

$$F = \frac{1}{2}(1 + Ae^{-(t/T)^n}). \tag{12}$$

Here, we obtain for the error-corrected logical qubit: $T = 24.2(2)$ ms and $n = 2.03(7)$, while for the best qubit we find: $T = 17.3(2)$ ms and $n = 2.09(7)$. For the encoded qubit with majority voting we obtain: $T = 13.7(1)$ ms and $n = 2.37(8)$.

To get a better understanding of quantum error correction and the projection of errors in the experiments with stabilizer measurements at half the free evolution time in Fig. 4d we turn to numerical Monte Carlo simulations, see Fig. 10 for details and results.

Supplementary Note 2: Theoretical analysis: error probabilities

The probability to detect no error ($P^{(0)}$) is the sum of the probability to have no error (no qubits flipped) or three errors (all qubits flipped) and is described by:

$$P^{(0)} = (1 - p_{\mathrm{tot}}^{(1)})(1 - p_{\mathrm{tot}}^{(2)})(1 - p_{\mathrm{tot}}^{(3)}) + p_{\mathrm{tot}}^{(1)}p_{\mathrm{tot}}^{(2)}p_{\mathrm{tot}}^{(3)}, \tag{13}$$

where $p_{\mathrm{tot}}^{(i)}$ is the error probability for qubit i. The probability to detect an error on one of the three qubits ($P^{(i)}$) is the probability to have an error on qubit i, or an error on both of the other qubits, which for example for qubit 1 is described by:

$$P^{(1)} = p_{\mathrm{tot}}^{(1)}(1 - p_{\mathrm{tot}}^{(2)})(1 - p_{\mathrm{tot}}^{(3)}) + (1 - p_{\mathrm{tot}}^{(1)})p_{\mathrm{tot}}^{(2)}p_{\mathrm{tot}}^{(3)}. \tag{14}$$

With finite input error probability $p_{\mathrm{in}}^{(i)}$ for qubit i (errors already present in the initially prepared state), the total error as function of the applied error probability p_e, becomes:

$$p_{\mathrm{tot}}^{(i)} = p_{\mathrm{in}}^{(i)} + p_e - 2p_{\mathrm{in}}^{(i)}p_e \tag{15}$$

Finally we can take imperfect ancilla readout into account and obtain the probability to detect one of the error outcomes $P_{\mathrm{D}}^{(i)}$ (i=0 for no detected error) as function of the applied error p_e:

$$P_{\mathrm{D}}^{(0)} = P^{(0)}F^2 + (P^{(1)} + P^{(3)})F(1 - F) + P^{(2)}(1 - F)^2 \tag{16}$$

$$P_{\mathrm{D}}^{(1)} = P^{(1)}F^2 + (P^{(0)} + P^{(2)})F(1 - F) + P^{(3)}(1 - F)^2 \tag{17}$$

$$P_{\mathrm{D}}^{(2)} = P^{(2)}F^2 + (P^{(1)} + P^{(3)})F(1 - F) + P^{(0)}(1 - F)^2 \tag{18}$$

$$P_{\mathrm{D}}^{(3)} = P^{(3)}F^2 + (P^{(0)} + P^{(2)})F(1 - F) + P^{(1)}(1 - F)^2 \tag{19}$$

The XX stabilizers in the encoded state tomography (Fig. 2) detect errors present in the encoded state, we obtain:

$$P^{(0)} =\langle\frac{1}{4}(1 + X_1, X_2, I_3)(1 + X_1, I_2, X_3)\rangle = \frac{1}{4}(1 + \langle X_1, X_2, I_3\rangle + \langle I_1, X_2, X_3\rangle + \langle X_1, I_2, X_3\rangle) = 0.785(2) \tag{20}$$

$$P^{(1)} =\langle\frac{1}{4}(1 - X_1, X_2, I_3)(1 - X_1, I_2, X_3)\rangle = \frac{1}{4}(1 - \langle X_1, X_2, I_3\rangle + \langle I_1, X_2, X_3\rangle - \langle X_1, I_2, X_3\rangle) = 0.060(2) \tag{21}$$

$$P^{(2)} =\langle\frac{1}{4}(1 - X_1, X_2, I_3)(1 + X_1, I_2, X_3)\rangle = \frac{1}{4}(1 - \langle X_1, X_2, I_3\rangle - \langle I_1, X_2, X_3\rangle + \langle X_1, I_2, X_3\rangle) = 0.083(2) \tag{22}$$

$$P^{(3)} =\langle\frac{1}{4}(1 + X_1, X_2, I_3)(1 - X_1, I_2, X_3)\rangle = \frac{1}{4}(1 + \langle X_1, X_2, I_3\rangle - \langle I_1, X_2, X_3\rangle - \langle X_1, I_2, X_3\rangle) = 0.071(2) \tag{23}$$

which are uncorrected for qubit readout. These results can be translated to the input errors, as these outcomes refer to Eqs. 13&14 with no additional applied error p_e, giving $p_{\rm in}^{(1)} = 0.064(2)$, $p_{\rm in}^{(2)} = 0.091(2)$, $p_{\rm in}^{(3)} = 0.077(2)$.

Using these values we estimate the expected total error detection probabilities $P_{\rm D}^{(0)}$, $P_{\rm D}^{(1)}$, $P_{\rm D}^{(2)}$ and $P_{\rm D}^{(3)}$ as function of applied error probability p_e according to Eqs. 13-19. The expected error-dependent QEC measurement outcomes are shown by the solid lines in the inset of Fig. 3b.

Error syndrome assignment

For the different error assignments, the asymmetry in the ancilla readout complicates the error detection curves: the QEC measurement fidelity is dependent on the error probability. If, for instance, both stabilizer measurements giving +1 are assigned to $\{|1\rangle_{\rm a}, |1\rangle_{\rm a}\}$, Eqs. 16-19 become:

$$P_{11}^{(0)} = P^{(0)}F_1^2 + (P^{(1)} + P^{(3)})F_1(1 - F_0) + P^{(2)}(1 - F_0)^2 \tag{24}$$

$$P_{11}^{(1)} = P^{(1)}F_1F_0 + P^{(0)}F_1(1 - F_1) + P^{(2)}F_0(1 - F_0) + P^{(3)}(1 - F_1)(1 - F_0) \tag{25}$$

$$P_{11}^{(2)} = P^{(2)}F_0^2 + (P^{(1)} + P^{(3)})F_0(1 - F_1) + P^{(0)}(1 - F_1)^2 \tag{26}$$

$$P_{11}^{(3)} = P^{(3)}F_1F_0 + P^{(0)}F_1(1 - F_1) + P^{(2)}F_0(1 - F_0) + P^{(2)}(1 - F_1)(1 - F_0) \tag{27}$$

All error detection curves for the four error assignments using similar equations are plotted in Supplementary Fig. 8.

Multiple rounds of error correction, Fig. 4b

For multiple rounds we now calculate the average input error $p_{\rm in}^{\rm (avg)}$ from the detection probability for no additional applied error ($p_e = 0$). We simplify Eq. 13 to

$$P^{(0)} = 1 - 3p_{\rm tot}^{\rm (avg)} + 3(p_{\rm tot}^{\rm (avg)})^2 \tag{28}$$

and use Eq. 24 to obtain the following average input error for round 1: $p_{\rm in}^{\rm (avg)} = 0.092(1)$ and for round 2: $p_{\rm in}^{\rm (avg)} = 0.086(1)$. The resulting curves according to Eq. 24 are shown in the inset of Fig. 4b.

Supplementary Note 3: Qubit readout calibration

To obtain best estimates for the actual states, the results are corrected for the fidelity of the gates used in the final readout (tomography). We distinguish between reading out single- two- and three-qubit expectation values.

For a single qubit i that is initialized and readout immediately, the measured expectation value $\langle Z_i\rangle$ is set by the initialization fidelity of the nitrogen spin ($F_{\rm N} = 0.94(3)$) and by factors due to the initialization ($C_{{\rm init},Qi}$) and readout (C_{Q_i}) of the qubit. Because the initialization and readout consist of the same set of gates, we assume that $C_{{\rm init},Qi} = C_{Q_i}$ for this experiment and obtain:

$$\langle Z_i\rangle = F_{\rm N}C_{Q_i}^2, \tag{29}$$

from which a readout correction factor $1/C_{Q_i}$ can be determined.

To calibrate the multi-qubit readouts we initialize the three qubits in separable states. For example, for state $|000\rangle$, the measured three-qubit expectation value $\langle Z_1Z_2Z_3\rangle$ is set by the nitrogen initialization F_{N}, by factors $C_{\mathrm{init},Qi}$ due to the individual initialization fidelities of the three-qubits and by a factor C_{Q_1,Q_2,Q_3} due to the three-qubit readout:

$$\langle Z_1Z_2Z_3\rangle = F_{\mathrm{N}}C_{\mathrm{init},Q1}C_{\mathrm{init},Q2}C_{\mathrm{init},Q3}C_{Q_1,Q_2,Q_3} \to C_{Q_1,Q_2,Q_3} = \frac{\langle Z_1Z_2Z_3\rangle}{F_{\mathrm{N}}C_{\mathrm{init},Q1}C_{\mathrm{init},Q2}C_{\mathrm{init},Q3}}. \tag{30}$$

This equation assumes that the initialization errors, other than those due to the nitrogen initialization, are uncorrelated. The initialization fidelities are obtained using the single-qubit expectation values and single qubit C_{Q_i} for the corresponding qubit, i.e. for qubit 1:

$$\langle Z_1I_2I_3\rangle = F_{\mathrm{N}}C_{\mathrm{init},Q1}C_{Q_1} \to C_{\mathrm{init},Q1} = \frac{\langle Z_1I_2I_3\rangle}{F_{\mathrm{N}}C_{Q_1}} \tag{31}$$

with C_{Q_1} from Eq. 29. In a similar way, the two-qubit readout is calibrated using two-qubit expectation values of two- and three-qubit states. We obtain the following values:

$$\begin{array}{lll} C_{Q_1} = 0.95(1) & C_{Q_1Q_2} = 0.94(2) & C_{Q_1Q_2Q_3} = 0.92(5) \\ C_{Q_2} = 0.94(1) & C_{Q_1Q_3} = 0.88(4) & \\ C_{Q_3} = 0.95(1) & C_{Q_2Q_3} = 0.90(2) & \end{array}$$

which are used to calibrate the final readouts for tomography. Note that the uncertainty in the readout calibration potentially creates a small systematic error (a rescaling of all y-axes). For this reason we also provide all raw (uncalibrated) data for the error correction in Supplementary Fig. 11.

Although our data do not yield a rigorous value of the two-qubit gate fidelity, the qubit readout fidelities derived here, $F = (1 + C_{Q_i})/2 \approx 0.97$ give an indication of the two-qubit gate fidelity as readout consists of two single qubit gates and one two-qubit gate.

SUPPLEMENTARY REFERENCES

[1] Taminiau, T. H., Cramer, J., van der Sar, T., Dobrovitski, V. V. & Hanson, R. Universal control and error correction in multi-qubit spin registers in diamond. *Nature Nanotech.* **9**, 171-176 (2014).

[2] Robledo, L. *et al.* High-fidelity projective read-out of a solid-state spin quantum register. *Nature* **477**, 574-578 (2011).

[3] Blok, M. S. *et al.* Manipulating a qubit through the backaction of sequential partial measurements and real-time feedback. *Nature Phys.* **10**, 189-193 (2014).
